# A Multi-Perspective Proximity View on the Dynamic Head Region of the Ribosomal 40S Subunit

**DOI:** 10.3390/ijms222111653

**Published:** 2021-10-28

**Authors:** Kerstin Schmitt, Alina-Andrea Kraft, Oliver Valerius

**Affiliations:** Department of Molecular Microbiology and Genetics, Institute of Microbiology and Genetics, Göttingen Center for Molecular Biosciences (GZMB), Georg-August-University Göttingen, 37077 Göttingen, Germany; kschmit1@gwdg.de (K.S.); alinaandrea.kraft@stud.uni-goettingen.de (A.-A.K.)

**Keywords:** biotin identification (BioID), Asc1/RACK1, Rps2/uS5, Rps3/uS3, Rps20/uS10, Rpl5/uL18, ribosome-associated protein quality control (RQC), Split-TurboID

## Abstract

A comparison of overlapping proximity captures at the head region of the ribosomal 40S subunit (*hr40S*) in *Saccharomyces cerevisiae* from four adjacent perspectives, namely Asc1/RACK1, Rps2/uS5, Rps3/uS3, and Rps20/uS10, corroborates dynamic co-localization of proteins that control activity and fate of both ribosomes and mRNA. Co-locating factors that associate with the *hr40S* are involved in (i) (de)ubiquitination of ribosomal proteins (Hel2, Bre5-Ubp3), (ii) clamping of inactive ribosomal subunits (Stm1), (iii) mRNA surveillance and vesicular transport (Smy2, Syh1), (iv) degradation of mRNA (endo- and exonucleases Ypl199c and Xrn1, respectively), (v) autophagy (Psp2, Vps30, Ykt6), and (vi) kinase signaling (Ste20). Additionally, they must be harmonized with translation initiation factors (eIF3, cap-binding protein Cdc33, eIF2A) and mRNA-binding/ribosome-charging proteins (Scp160, Sro9). The Rps/uS-BioID perspectives revealed substantial Asc1/RACK1-dependent *hr40S* configuration indicating a function of the β-propeller in context-specific spatial organization of this microenvironment. Toward resolving context-specific constellations, a Split-TurboID analysis emphasized the ubiquitin-associated factors Def1 and Lsm12 as neighbors of Bre5 at *hr40S*. These shuttling proteins indicate a common regulatory axis for the fate of polymerizing machineries for the biosynthesis of proteins in the cytoplasm and RNA/DNA in the nucleus.

## 1. Introduction

Comprehension of the cellular integration of ribosomes requires an understanding of dynamic interactions between ribosome and non-ribosomal components as well as between two proximal ribosomes (e.g., collided or aggregated ones). These interactions may be temporary and dynamic in regard to specific cellular contexts. An exposed ribosomal contact point is the head region of the 40S ribosomal subunit (*hr40S*). In recent years, different proximity labeling techniques have emerged and are continuously being optimized, which are well suited for the identification of non-ribosomal and ribosomal components entering within short distance of this site [1]. The biotin IDentification (BioID) approach makes use of a promiscuous biotin ligase that is fused to a protein of interest and causes in vivo labeling of proximal proteins with biotin [2]. Biotinylated proteins are enriched from cell lysates using affinity matrices such as Streptavidin or Strep-Tactin^®^ and can be identified using targeted validation like Western blot experiments or unbiased approaches like liquid chromatography-mass spectrometry (LC-MS). This methodology is especially convenient for studying dynamic contact sites of large protein or ribonucleoprotein complexes. For instance, factors involved in the supply or regulation of biosynthetic machineries associate context specifically to these sites, and due to their weak and/or transient binding, are generally difficult to identify with pull-down experiments. The BioID methodology does not rely on sustained protein binding within cell lysates, but rather on cellular proximity during culture growth. In previous work, we have established a quantitative BioID approach with *stable isotope labeling with amino acids in cell culture* (SILAC-BioID) to start identifying proteins that co-localize with the WD40-repeat and β-propeller protein Asc1 in *S. cerevisiae*, the conserved homolog of RACK1 in higher eukaryotes [3]. Asc1/RACK1 resides at the *hr40S* close to the mRNA exit tunnel and is linked to the mRNA entry channel via binding to the extended C-terminal arm of Rps3 [4]. Asc1/RACK1 is exposed to the cytoplasm and faces regulators of the initiation of mRNA translation and signal transduction components [3,5,6,7]. It provides resistance against and viability in challenging growth conditions such as nutrient depletion, osmotic stress, temperature shock or cell wall burden and is required for multicellular development in higher fungi [8,9,10]. In metazoa, RACK1 is crucial for embryogenesis [11,12], and aberrant expression levels of RACK1 are associated with poor clinical outcome of different types of cancers [13]. RACK1 is involved in cellular signaling and contributes to fundamental cellular processes such as cell proliferation, cell migration, apoptosis, and angiogenesis [13].

We previously identified proteins that had not been known for being close to ribosomes, specifically at the *hr40S*, such as Mbf1 and Def1, the E3-ubiquitin RING ligase Hel2, and the deubiquitination complex Bre5-Ubp3 [3]. In the meantime, it has been shown that Mbf1, in concert with Rps3 and Asc1, prevents ribosomal frameshifting at inhibitory CGA–CGA codon pairs [14]. Moreover, Mbf1 and its mammalian homolog EDF1 are present at colliding ribosomes near the mRNA entry channel upon stall-inducing conditions [15]. Def1 is involved in the degradation of stalled RNA polymerase II (RNAPII) within the nucleus [16]. Co-purification experiments showed that Def1 and RNAPII also interact with Bre5-Ubp3, and that Ubp3 mediates deubiquitination of RNAPII [17].

**Figure 1:**
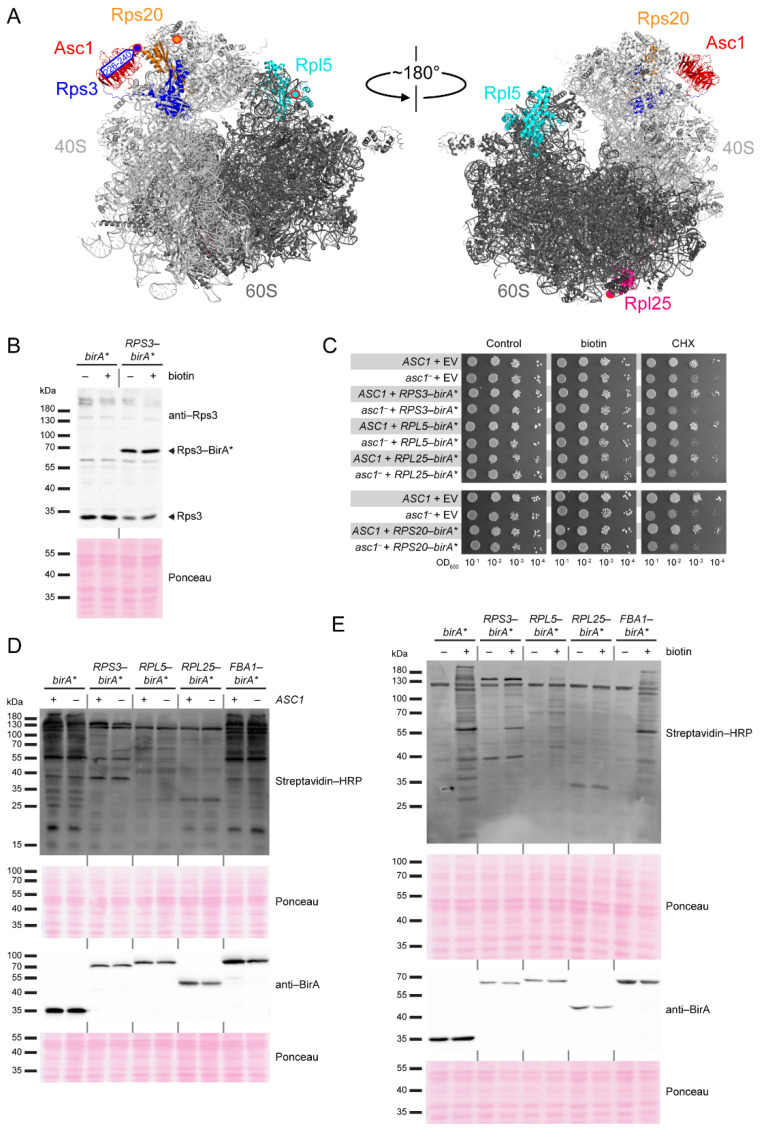
BirA* fusion protein expression and monitoring of biotinylation activity. (**A**) Structure of the *S. cerevisiae* 80S ribosome with Asc1 (red), Rps3 (blue), Rps20 (orange), Rpl5 (cyan), and Rpl25 (pink) highlighted. rRNA and proteins of the 40S subunit are colored in light gray and rRNA and proteins of the 60S subunit in dark gray. Since the proteins of interest were C-terminally fused to BirA*, the (approximate) position of each C-terminus is indicated with a red-rimmed circle filled with the respective color. For Rps3, the C-terminal amino acids R226-A240, which are structurally not resolved, are indicated with an arrow. Crystal structure data from PDB entry 4V88 [4] were visualized using the PyMOL molecular graphics system. (**B**) Protein extracts of *ASC1* wild-type cells expressing either free BirA* or Rps3-BirA* were separated by SDS polyacrylamide gel electrophoresis (PAGE) and proteins were blotted onto a nitrocellulose membrane. An Rps3-specific antibody [3] was used to detect native endogenous Rps3 and the Rps3-BirA* fusion protein. Respective signals are labeled and highlighted with an arrow. Strains were cultivated in the presence and absence of 10 µM biotin. (**C**) The growth of *ASC1* wild-type and *asc1*^−^ strains (RH2817 and RH3510, respectively) expressing RP-BirA* fusion proteins was monitored on YNB medium with 10 µM biotin or 0.05 µg/mL cycloheximide (CHX) in comparison to strains with the empty vector (EV). A YNB plate without additional supplements served as a control. Tenfold serial dilutions of the cell cultures were spotted on the plates, and the cells were incubated for 3 d without and 4 d with CHX, respectively. (**D**,**E**) *S. cerevisiae ASC1* wild-type and *asc1*^−^ strains (RH3493 and RH3520) expressing plasmid-borne free BirA*, Rps3-BirA*, Rpl5-BirA*, Rpl25-BirA*, or Fba1-BirA* were cultivated in liquid medium and used for the preparation of protein extracts. Streptavidin-HRP was used for visualization of biotinylated proteins and a BirA-specific antibody for the detection of both free BirA* and the fusion proteins. Ponceau staining served as a loading control. (**D**) Free BirA* and the fusion proteins were expressed in *ASC1* wild-type (+) and *asc1*^−^ strains (−), and biotin (final concentration 10 µM) was added to all cultures. (**E**) *ASC1* wild-type cells expressing the indicated proteins were cultivated in the presence (+) or absence (−) of 10 µM biotin in the medium.

Since Asc1 is considered a scaffold protein that organizes protein microenvironments and might also appear in a ribosome-unbound state, we wanted to (i) validate Asc1-proximity of proteins in the *hr40S* microenvironment and (ii) study the impact of Asc1-depletion on their association with the *hr40S*, and with that on the integrity of this ribosomal microenvironment. Toward this end, we recently performed a BioID experiment from a slightly shifted perspective using Rps2/uS5-BirA* instead of Asc1-BirA* [18]. This confirmed, e.g., the ribosomal localization of Def1 and also indicated Asc1-dependence of Bre5-Ubp3 association with the *hr40S* [18]. Here, we consolidated the overall picture with two further ribosomal perspectives at the *hr40S*, namely that of Rps3/uS3 and Rps20/uS10 (Figure 1A). We established a new control for the *hr40S* BioID experiments, namely Rpl5/uL18-BirA*. Rpl5 is a protein of the 60S ribosomal subunit that on the one hand is part of the common machinery, but on the other hand is located at a distal site within the complex. We therefore call it a *distal control*.

Overall, our multi-perspective proximity analysis describes a range of proteins occurring at the head region close to the center of initiation of translation. Several factors have an impact on ubiquitin-mediated ribosome disposition linked to cellular signaling. The multitude of ribosomes within one cell and the high number of cells taken for a BioID experiment, lead to a cross-section of identified proteins that localize within this dynamic microenvironment with context-specific *hr40S* constellations. For the dissection of different protein constellations in ongoing studies, we established here the Split-TurboID approach for the first time for *S. cerevisiae* and identified proteins that are specific for an *hr40S* constellation that contains Bre5.

## 2. Results

### 2.1. Same Complex but Distal Site: A New Reference Point for hr40S SILAC-BioID Experiments

Appropriate controls serving as relative quantification reference points have to be chosen for the faithful identification of co-localizing proteins with proximity label/MS. With that, false positive evaluation of naturally biotinylated proteins or proteins labeled by stray activity, e.g., of an untethered BirA*-fusion protein moiety, can be avoided. In previous SILAC-BioID experiments, we used a wild-type yeast strain as well as a strain expressing a free BirA* ligase as negative controls. However, the latter one can cause substantial background biotinylation that might mask true neighbors of the bait-BirA* fusion protein [18]. This can be especially detrimental when the cellular abundance of the BirA*-bait protein is significantly less than that of free BirA*. In this case, the proximity-dependent biotinylation through the bait-BirA* fusion protein can be less than the unspecific background biotinylation through free BirA*. Alternatively, additional controls can be established with BirA* fused to proteins that are (i) expected to be functionally and regionally unrelated to the bait protein or (ii) members of the same complex as the bait protein but localizing apart at a distal site. Therefore, we tested BirA* fusions to the C-termini of the following proteins: (i) fructose 1,6-bisphosphate aldolase Fba1, an enzyme involved in glycolysis and gluconeogenesis, and (ii) Rpl5/uL18 and Rpl25/uL23, two proteins of the large 60S ribosomal subunit (Figure 1A). These two ribosomal proteins (RPs) appeared promising since comparable GFP fusion chimera have earlier been proven functional and applicable with respect to the baits’ localization and function [19]. The bait-BirA* fusion proteins were expressed in yeast cells bearing the respective endogenous wild-type alleles of the bait proteins. The abundance ratios of BirA*-fused and endogenous RPs were representatively analyzed for Rps3 with Western blot experiments (Figure 1B). The levels of endogenous untagged Rps3 decreased relative to the additional expression of plasmid-borne Rps3-BirA*, attributable to the *excess ribosomal protein quality control* (ERISQ) [20]. The expression of the RP-BirA* fusion proteins does not compromise general growth on standard yeast minimal medium neither in the presence nor in the absence of additional biotin. Wild-type-like resistance against the mRNA translation inhibitor cycloheximide also verified natural ribosome resilience when RP-BirA* fusion proteins were expressed (Figure 1C). Asc1-deficient *asc1*^−^ cells became only marginally more sensitive to cycloheximide when they were expressing the RP-BirA* fusion proteins. We tested for the expression of the RP-BirA* fusion proteins and their biotinylation activities (Figure 1D). Stable expression of all BirA* fusion proteins was confirmed with Western blot experiments using a BirA-specific antibody. Depletion of Asc1 neither affected the abundance of the fusion proteins nor their overall biotinylation activity. As mentioned before, a high dosage of biotin causes strong overall protein biotinylation for cells expressing free BirA* (Figure 1E, [3,18]). The biotinylation activity of Fba1-BirA* was comparably strong to that of free BirA*, probably due to the high cellular protein abundance of Fba1 (Figure 1D,E, [21]). By contrast, only a slight increase in biotinylation activity was observed for Rpl25-BirA*. For Rps3-BirA* and Rpl5-BirA*, a substantial and similar increase in overall protein biotinylation was observed. Since biotin labeling by these two RP-BirA* fusion proteins seemed to be most similar among the tested constructs, Rpl5-BirA* was chosen as a distal reference control for SILAC quantification in further proximity mappings at the *hr40S* and is subsequently referred to as *distal control*.

### 2.2. Proximity Labeling with Biotin at the hr40S from the Rps3 Perspective

**Figure 2:**
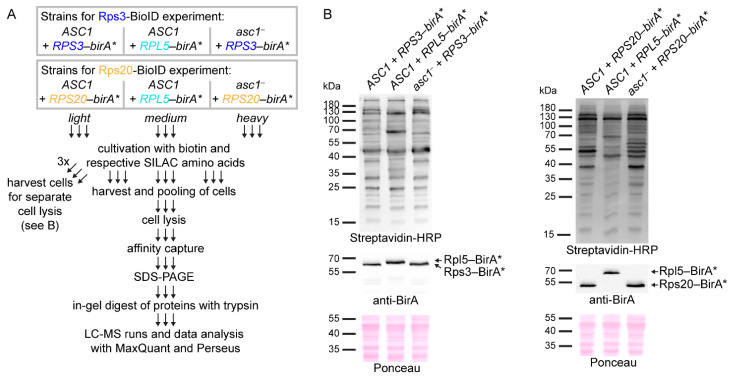
Workflow of the Rps3 and Rps20 proximity labeling MS experiments. (**A**) For the Rps3-BioID experiment, *ASC1* wild-type and *asc1*^−^ strains (RH3493 and RH3520, respectively) expressing plasmid-borne Rps3-BirA* were cultivated with *light* and *heavy* SILAC amino acids, respectively. As a negative control, an *ASC1* wild-type strain expressing Rpl5-BirA* was labeled with the *medium* SILAC amino acids. Equivalently, the Rps20-BioID experiment was performed using strains expressing Rps20-BirA* instead of Rps3-BirA*. Cells were cultivated in the presence of the respective SILAC amino acids and with 10 µM biotin. Cells of the individual cultures were harvested in the exponential growth phase and combined. An aliquot of the still separate cell cultures was taken for cell lysis for the Western blot experiments depicted in (**B**). The protein extracts of the pooled cells were used to enrich biotinylated proteins via affinity purification. Proteins were subjected to SDS-PAGE followed by in-gel digestion with trypsin. Peptides were analyzed with LC-MS, and data analysis was performed using the MaxQuant and Perseus software. (**B**) Cell lysates of the indicated strains were separated by SDS-PAGE and proteins transferred onto nitrocellulose membranes to detect RP-BirA* fusion proteins and biotinylated proteins using a BirA-specific antibody and Streptavidin-HRP, respectively. One of three replicates is shown representatively.

**Figure 3:**
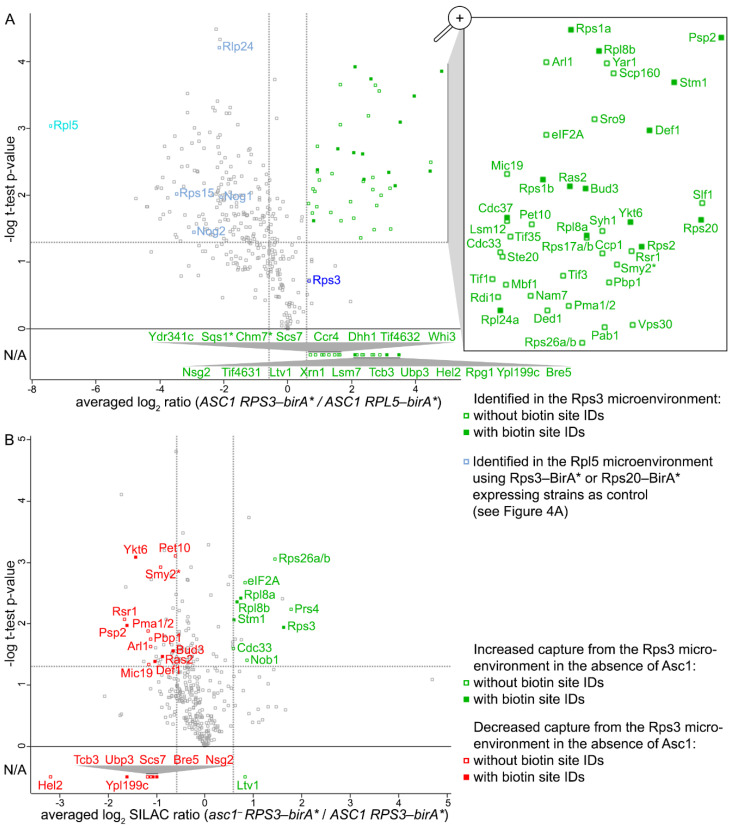
Proteins occurring proximal to Rps3 and their Asc1-dependence. Volcano plots show the averaged log_2_ SILAC ratios for (**A**) ASC1 + RPS3-birA* (*light* SILAC) to ASC1 + RPL5-birA* (*medium* SILAC) and (**B**) *asc1*^−^ + RPS3-birA* (*heavy* SILAC) to ASC1 + RPS3-birA* (*light* SILAC) on the x-axes and the -log_10_ (*p*-value) of the t-test on the y-axes. Gray dotted threshold lines indicate a *p*-value of 0.05 (horizontal) and a log_2_ SILAC ratio of 0.585 or −0.585 (vertical). Proteins with two instead of three quantification values (w/o *p*-values) that passed the threshold of two values ≥ 0.585 are depicted below the horizontal 0-value line with their averaged log_2_ SILAC ratios (L/M in A and H/L in B). Not applicable (N/A) on the y-axis indicates that no *t*-test was performed for these proteins and thus no *p*-value is available. For proteins highlighted with an asterisks (*), no information about their protein abundance was obtained from the proteome data. For all other proteins that are highlighted in red or green, at least one quantification value was obtained from the proteome analysis. Gray squares represent proteins not passing the set criteria for proximity (**A**) or Asc1-dependence (**B**). For details see Tables S1–S5.

For an Rps3-BirA* SILAC-BioID experiment, an RPS3-birA* fusion gene-bearing strain was grown with *light* and an RPL5-birA*-bearing strain (the newly established distal control) with *medium* isotope-labeled SILAC amino acids. Additionally, a *heavy* isotope-labeled *asc1*^−^ strain bearing RPS3-birA* was included to analyze the Asc1-dependence of the captured Rps3 microenvironment (Figure 2A). After separate culture growth, similar numbers of cells of the three cultures were pooled and lysed in one batch under denaturing conditions (4% SDS). Aliquots were taken from the separate cultures for cell lysis to confirm stable expression and biotinylation activity of bait-BirA* fusion proteins by Western blot experiments (Figure 2B). Biotinylated proteins from the lysate of the cell pool were enriched with Strep-Tactin^®^ gravity flow columns, digested with trypsin, and the resulting peptides were analyzed by LC-MS (Figure 2A). Three independent biological replicates were analyzed, and the MS data were searched against an *S. cerevisiae*-specific protein database (derived from UniProt) for peptide/protein identification and relative quantification by using the MaxQuant software [22]. The quantitative search result data were further analyzed with the Perseus software [23] according to the workflow summarized in Table S1. SILAC ratios were logarithmized and proteins with valid enrichment quantification values for all three replicates were further considered (360 proteins). The BioID input samples (total proteomes) were additionally analyzed with LC-MS to account for possible expression variances within the different yeast strains. The BioID-enrichment ratios were then normalized to these proteome ratios, leading to proteome-corrected BioID-enrichment values. Based on low cellular expression, some proteins with BioID-enrichment data were not identified/quantified from the proteome input samples. For these proteins, data analysis proceeded on the basis of the enrichment ratios only. A one-sample t-test was performed for the proteome-corrected enrichment ratios of the ASC1 + RPS3-birA*/ASC1 + RPL5-birA* comparison with a *p*-value threshold of 0.05. Additionally, proteins were filtered for at least 1.5-fold enrichment (log_2_ SILAC ratios ≥ 0.585). A total of 42 proteins passed these thresholds and were thus considered as occurring proximal to Rps3 (Figure 3A, highlighted in green in the zoomed in box; Table S2).

Among these proteins were seven ribosomal proteins (Rps1a/b, Rps2, Rps17a/b, Rps20, Rps26a/b, Rpl8a/b, and Rpl24a), the ribosome clamping factor Stm1, translation initiation factors (Cdc33, Tif1, Tif3, Tif35, eIF2A), the poly(A)-binding protein Pab1, the Pab1-binding protein Pbp1, mRNA-binding proteins (Sro9, Scp160, and Slf1), and the Rps3-chaperone Yar1, indicating that the native Rps3 microenvironment was captured. Other proteins identified from previous BioID experiments with Asc1-BirA* and Rps2-BirA* recurred with Rps3-BirA* here, namely, Def1, Lsm12, Mbf1, and the two paralogs Smy2 and Syh1, and Vps30 [3,18]. Vps30, a subunit of the phosphatidylinositol 3-kinase (PI3K) complexes I and II, was identified in the Rps2-BirA* microenvironment only in the absence of Asc1 [18]. However, here we observed Vps30 proximal to Rps3-BirA* also in the presence of Asc1. The PI3K complexes I and II are involved in autophagosome formation and vacuolar protein sorting [24].

Also, Psp2, a protein that has recently been described to be involved in autophagy regulation in yeast, was identified within the Rps3-BirA* microenvironment. Upon nitrogen starvation, Psp2 binds 5′UTRs of mRNAs encoding the autophagy factors Atg1 and Atg13, and by this promotes the initiation of their translation through interaction with components of the eIF4F complex [25]. Further, the R-SNARE Ykt6 required for autophagosome-vacuole fusion [26,27] and the small GTPase Arl1 involved in autophagosome formation and fusion with the vacuole [28] were found proximal to Rps3-BirA*. Some further captured proteins are involved in signal transduction, e.g., the upstream mitogen-activated protein 4 kinase (MAP4K) Ste20 and the GTP-binding protein Ras2.

Beyond that, we also identified 274 proteins with only two out of three valid quantification values. Among them were an additional 19 proteins with at least 1.5-fold enrichment from the Rps3-BirA* samples (Figure 3A; Table S3). These were, e.g., the ubiquitin ligase Hel2, the deubiquitinase Ubp3 and its co-factor Bre5, and the translation initiation factor eIF3a (Rpg1) that have also been found proximal to the *hr40S* in our previous BioID experiments [3,18]. Ypl199c is another protein of this group. The Smr domain containing proteins Ypl199c and Cue2 are homologs of the *Caenorhabditis elegans* endonuclease NONU-1, and they have recently been shown to redundantly support nonstop mRNA decay [29]. To our knowledge, this is the first study indicating the localization of the putative endonuclease Ypl199c to the *hr40S* in *S. cerevisiae*.

### 2.3. The Asc1-Dependent Microenvironment of Rps3-BirA*

To reveal Asc1-dependent differences at the *hr40S*, we then quantitatively compared the Rps3-BirA* proximal proteins in the presence and absence of Asc1 by using *ASC1* wild-type and *asc1*^−^ strains (Figure 3B). Again, proteins with either three or two enrichment values were taken into account, and an averaged log_2_ difference of ≥ 0.585 or ≤ −0.585 was set as a threshold for Asc1-dependent Rps3-BirA* proximity (Table S1). A total of 19 proteins showed decreased and ten proteins showed increased capture in the absence of Asc1 (Figure 3B; Tables S4 and S5). These data confirmed a previously observed Asc1-dependence in the *hr40S* localization of Bre5-Ubp3 [18], a complex known to deubiquitinate Rps3 [30]. In addition, the E3-ubiquitin RING ligase Hel2 that ubiquitinates Rps3 at collided ribosomes and the putative endonuclease Ypl199c were less captured from the *asc1*^−^ strain. Reduced capture of the aforementioned autophagy factors Psp2, Arl1, and Ykt6 might indicate an impact of Asc1 on autophagy-related processes.

### 2.4. Another Step Ahead: The hr40S Proxiome from the Rps20-BirA* Perspective

**Figure 4:**
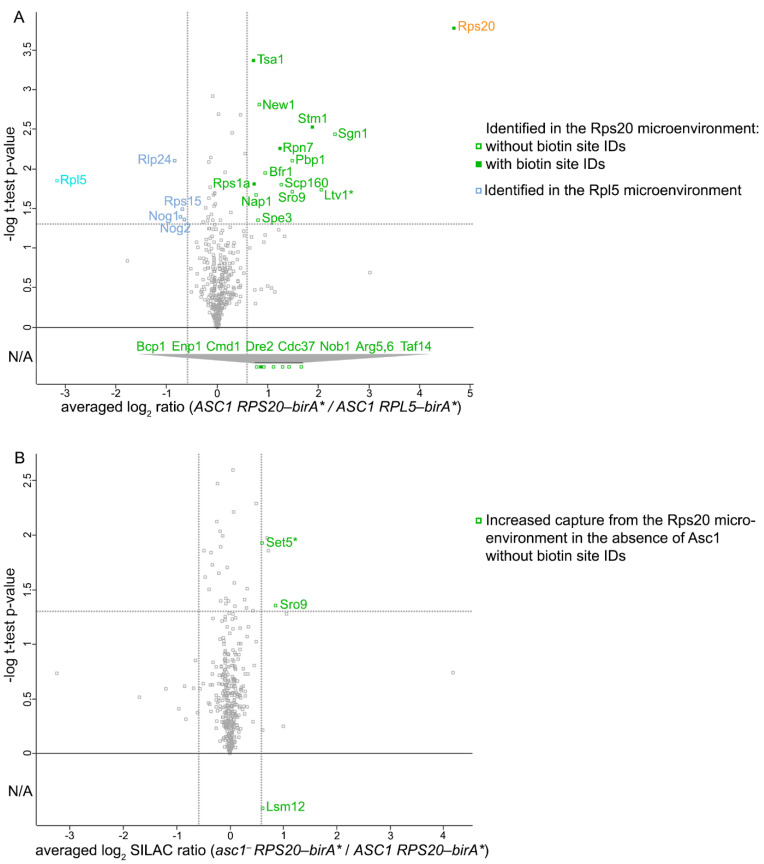
Proteins occurring proximal to Rps20 and their Asc1-dependence. Volcano plots show the averaged log_2_ SILAC ratios for (**A**) ASC1 RPS20-birA* (*light* SILAC) to ASC1 RPL5-birA* (*medium* SILAC) and (**B**) *asc1*^−^ RPS20-birA* (*heavy* SILAC) to ASC1 RPS20-birA* (*light* SILAC) on the x-axes and the -log_10_ (*p*-value) of the t-test on the y-axes. Gray dotted threshold lines indicate a *p*-value of 0.05 (horizontal) and a log_2_ SILAC ratio of 0.585 or −0.585 (vertical). Proteins with two instead of three quantification values (w/o *p*-values) that passed the threshold of two values ≥ 0.585 are depicted below the horizontal 0-value line with their averaged log_2_ SILAC ratios (L/M in (**A**) and H/L in (**B**)). Not applicable (N/A) on the y-axis indicates that no t-test was performed for these proteins and thus no *p*-value is available. For proteins highlighted with an asterisks (*), no information about their protein abundance was obtained from the proteome data. For all other proteins that are highlighted in red or green, at least one quantification value was obtained from the proteome analysis. Gray squares represent proteins not passing the set criteria for proximity (**A**) or Asc1-dependence (**B**). For details see Tables S1 and S6–S9.

For the results described thus far, Rpl5-BirA* served as a good control, so we held on to it for another SILAC-BioID experiment at the *hr40S* with Rps20/uS10-BirA* (Figure 1A). We observed stable expression of Rps20-BirA* and no detrimental effects on colony growth and translational stress tolerance (Figure 1C and Figure 2B). The workflow of the SILAC-BioID experiment and the data analysis was essentially identical to that described before (Figure 2A and Table S1; for minor variations see materials and methods). A total of 401 proteins with quantification values from all three replicates were identified and a further 338 proteins with two quantification values. Regarding the proteins with three quantification values, 14 of them were specifically enriched with Rps20-BirA*, including the bait protein itself (≥1.5-fold enrichment, *p*-value threshold of 0.05; Figure 4A; Table S6). Eight additional proteins appeared that had only two enrichment ratio values, but at least 1.5-fold enrichment (Table S7). Seven of all these proteins were known from previous SILAC-BioID experiments as being proximal to the *hr40S*. Furthermore, the known Rps20 co-localizing [NU+] prion formation protein New1 was identified. New1 is very similar to the translation elongation factor eEF3 with regard to its domain architecture as well as its binding to the 80S ribosome [31]. New1 is important for translation termination and/or ribosome recycling at stop codons that are preceded by lysine and arginine codons [31].

Thioredoxin peroxidase Tsa1 was another protein within the proximity of Rps20-BirA*. Tsa1 has a described, but thus far unspecified, ribosomal localization and appears to serve as an antioxidant for translating ribosomes [32]. The enzyme Spe3 which catalyzes the synthesis of the polyamine spermidine was also identified. Polyamines bind nucleic acids and promote the efficiency and fidelity of translation [33]. Spermidine is a substrate for the essential hypusine modification of eIF5A, a translation factor required for the synthesis of proteins containing polyproline sequences and for the termination of translation [33]. The absence of Asc1 only caused minor changes within the Rps20-BirA* microenvironment: Sro9, Lsm12, and Set5 showed increased capture (Figure 4B; Tables S8 and S9) and for Set5, no proteome values were obtained for the input normalization, indicating that the enrichment could also have been due to increased cellular protein abundance in the absence of Asc1.

### 2.5. Toward the Bre5-Specific hr40S Microenvironment with Split-TurboID

**Figure 5:**
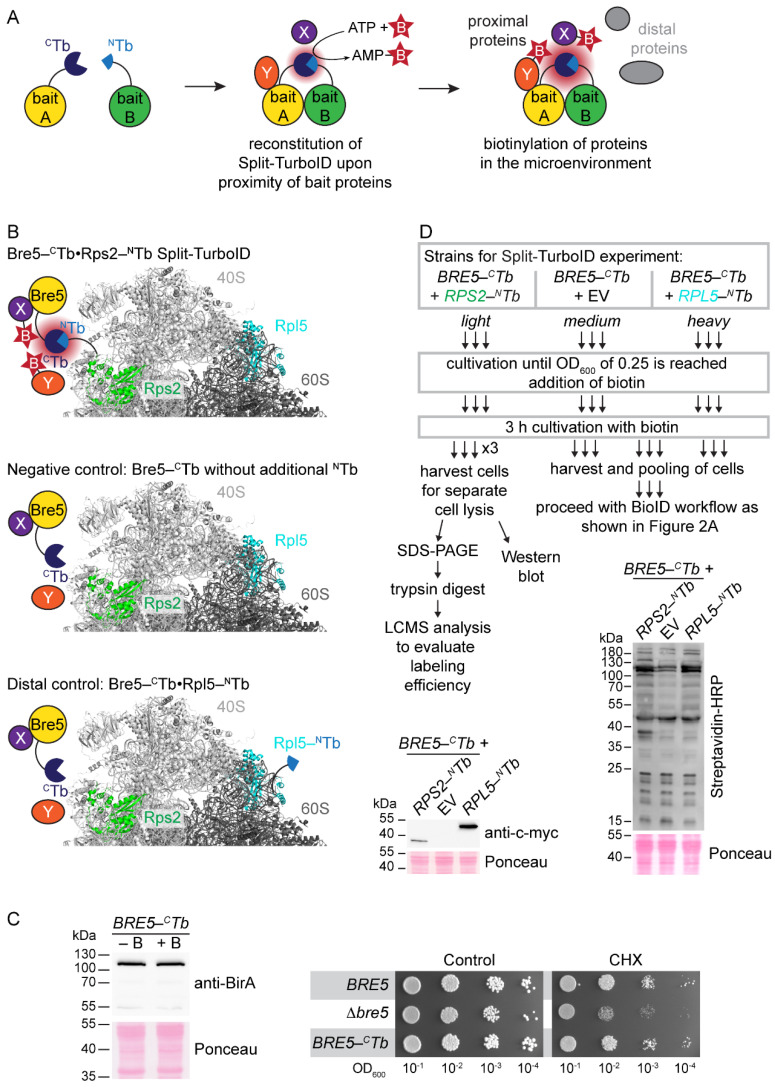
Split-TurboID experiment to analyze the Bre5 microenvironment during co-localization with Rps2. (**A**) Principle of Split-TurboID. Upon co-localization of the bait proteins, the Split-TurboID halves reconstitute to a functional biotin ligase. The enzyme catalyzes the formation of biotinyl-AMP that reacts with primary amines of proteins in the proximity (dubbed x and y). (**B**) Bre5-^C^Tb and Rps2-^N^Tb were co-expressed to biotinylate proteins in their common microenvironment. As a negative control, Bre5-^C^Tb is expressed in the absence of any ^N^Tb to account for mild biotinylation activity of ^C^Tb. As a distal control, Bre5-^C^Tb was co-expressed with Rpl5-^N^Tb. They are supposed to locate at distal sites at the ribosome and residual biotinylation activity must be either unspecific or originate at a microenvironment apart from the *hr40S*. (**C**) Expression of Bre5-^C^Tb in the presence and absence of additional biotin (+B/−B) was validated in a Western blot experiment using a BirA-specific antibody. A drop dilution assay confirmed the functionality of the Bre5-^C^Tb fusion protein. Tenfold serial dilutions of the wild-type, the ∆*bre5* and the *BRE5-^C^Tb* strain were spotted on YNB plates containing 0.05 µg/mL cycloheximide (CHX). YNB plates served as a growth control. Plates were incubated for 3 d at 30 °C. (**D**) The Bre5-Rps2 Split-TurboID workflow: Strains were cultivated with SILAC amino acids as indicated until the cultures reached an OD_600_ of 0.25. After addition of biotin, incubation was continued for a further 3 h. Cells were harvested and combined. An aliquot of the still separate cell cultures was taken for cell lysis to obtain samples for the depicted Western blot experiments. Stable expression of Rps2-^N^Tb and Rpl5-^N^Tb was verified using an antibody against the myc-tag of the Tb-halves. Bre5-^C^Tb is not visible according to its significantly lower cellular abundance compared to the ribosomal proteins. Cellular biotinylation activity was evaluated using Streptavidin-HRP. The cell lysates were further used to evaluate the efficiency of protein labeling with 2nSILAC by LC-MS analysis. The remaining cells of the main cultures were pooled for preparation of cell lysates, and the samples were further processed as described for the BioID workflow in Figure 2A.

Thus far, our BioID survey has provided a cross section of a dynamic *hr40S* microenvironment where the ribosomal proteins Asc1, Rps2, Rps3, and Rps20 encounter a variety of non-ribosomal proteins, many of them putatively present there within a specific ribosomal context. Toward resolving context-specific protein proximities at the *hr40S*, we extended our analysis to Split-BioID experiments [34,35,36]. For a Split-BioID experiment, a biotin ligase is split into two catalytically inactive halves that are genetically fused to two bait proteins (Figure 5A). Upon co-localization of these bait proteins, a functional biotin ligase is reconstituted that biotinylates proteins within their common proximal microenvironment. Here, this methodology was adapted to *S. cerevisiae* for the first time with split halves of the BirA* variant TurboID, which has a stronger catalytic activity than BirA* [34]. Thus, short incubation times with biotin are sufficient to obtain pronounced protein biotinylation. To test whether Split-TurboID is generally applicable in yeast, we initially fused the N- and C-terminal TurboID halves (^N^Tb and ^C^Tb) to the C-termini of Asc1 and Rps2. Both proteins are highly abundant in yeast cells, their C-termini are proximal to each other and should therefore induce biotinylation activity through reconstitution of Split-TurboID. Additionally, we tested the free ^N^Tb and ^C^Tb halves. To confirm their expression with Western blot experiments using a myc-specific antibody, all constructs also contained a C-terminal myc tag; with Streptavidin-HRP we then monitored biotinylated proteins from cell lysates (Figure S1): Both combinations, Asc1-^C^Tb•Rps2-^N^Tb and Asc1-^N^Tb•Rps2-^C^Tb, resulted in substantial biotinylation activity indicating the reconstitution of a complemented TurboID. We also observed that the ^C^Tb on its own possesses low residual biotinylation activity. Moreover, free ^N^Tb and ^C^Tb halves reassemble to form a functional biotin ligase if expressed simultaneously. When one Tb-half was fused to either Asc1 or Rps2 and the other one was expressed individually without bait, substantial biotinylation was observed. Overexpression of Split-TurboID halves can lead to unspecific reconstitution of TurboID and should be avoided [37].

Previous BioID experiments with Asc1-, Rps2-, and Rps3-BirA* established Bre5, co-factor of the deubiquitinase Ubp3, as a protein that localizes to the *hr40S*. Bre5-Ubp3 plays a key role in ribophagy and stress granule formation [38], but also acts in the nucleus [17]. The complex also regulates anterograde and retrograde transport between the endoplasmic reticulum and the Golgi apparatus [39]. Thus, Bre5 is supposed to come across many different proteins in different cellular contexts. Here, we performed an *hr40S*-specific Bre5-Split-TurboID experiment to characterize its ribosomal microenvironment. Expression levels of Bre5 are far below that of RPs [21]. We expressed Bre5-^C^Tb from its genomic locus with its natural promoter to avoid overexpression and combined it with plasmid-borne *Rps2-^N^Tb*. Bre5-^C^Tb and Rps2-^N^Tb are supposed to form a functional biotin ligase when both bait proteins co-localize at the *hr40S* (Figure 5B). To account for the mild background biotinylation activity of ^C^Tb, a strain expressing Bre5-^C^Tb only (complemented with an empty vector, EV) was used as a negative control (*BRE5-^C^Tb*•EV). Again, we considered to use Rpl5 for a distal control, and thus included a Bre5-^C^Tb strain transformed by a plasmid expressing Rpl5-^N^Tb (*BRE5-^C^Tb*•*RPL5-^N^Tb*) in the experiment (Figure 5B).

Expression and functionality of Bre5-^C^Tb were confirmed by Western blot experiments using a BirA-specific antibody and by drop dilution growth assays on medium containing cycloheximide, respectively (Figure 5C). We performed a Split-TurboID experiment followed by LC-MS analysis essentially as described before for BioID experiments, including SILAC-labeling for relative enrichment quantification of proteins (Figure 5D). Here, we used yeast strains prototrophic for lysine and arginine and performed 2nSILAC [40]. We evaluated the labeling efficiency by determining the percentage of labeled peptides (based on annotated fragmentation spectra) in samples derived from the separate SILAC-labeled cultures (Tables S10 and S11). Over 98% of the peptides were correctly labeled in each sample, validating the 2nSILAC approach for the purpose of Split-TurboID experiments.

Taking advantage of the strong biotinylation activity of TurboID, the incubation time of cell cultures with biotin was reduced from overnight to 3 h. Western blot experiments with cell lysates from the separate cell cultures showed stable expression of Rps2-^N^Tb and Rpl5-^N^Tb (Figure 5D). Additionally, overall biotinylation activity was evaluated using Streptavidin-HRP. Co-expression of Bre5-^C^Tb and Rps2-^N^Tb resulted in an obvious increase in biotinylated proteins. For the distal control *BRE5-^C^Tb•RPL5-^N^Tb,* a slightly increased biotinylation was observed in comparison to the *BRE5-^C^Tb•*EV control.

**Figure 6:**
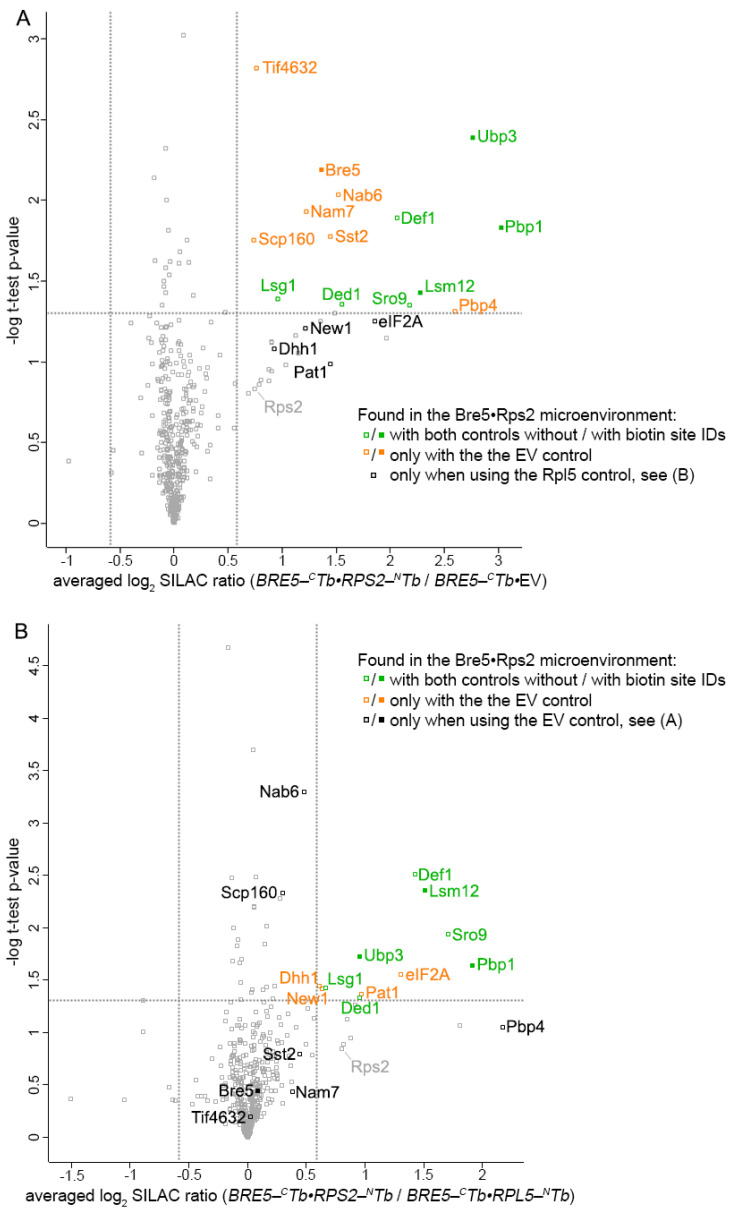
Proteins within a common microenvironment of Bre5 and Rps2. Volcano plots illustrate the averaged log_2_ SILAC ratios for (**A**) *BRE5-^C^Tb•RPS2-^N^Tb* (*light* SILAC) to *BRE5-^C^Tb•*EV (*medium* SILAC) and (**B**) *BRE5-^C^Tb•RPS2-^N^Tb* (*light* SILAC) to *BRE5-^C^Tb•RPL5-^N^Tb* (*heavy* SILAC) on the x-axes and the -log_10_(*p*-value) of the t-test on the y-axes. A *p*-value of 0.05 (horizontal) and a log_2_ SILAC ratio of 0.585 or −0.585 (vertical) are indicated with gray dotted threshold lines. Proteins found as significantly enriched from the Bre5-Rps2 microenvironment with both controls are highlighted in green. Proteins in orange passed these thresholds for only one of the comparisons. For all labeled proteins, eluate ratios were corrected on total proteome ratios (input normalization). Gray squares represent proteins not passing the set criteria for proximity. For details see Tables S12–S15.

All LC-MS data of the Split-TurboID experiment were analyzed similarly as described before for BioID experiments with regard to initial filtering steps, proteome-correction, and statistical validation (for details see Table S12). Using the Bre5-^C^Tb•EV strain as a negative control, 14 proteins were found as significantly captured from the Bre5-Rps2 microenvironment (Figure 6A; Tables S13 and S15), and 11 proteins with the distal control Bre5-^C^Tb•Rpl5-^N^Tb (Figure 6B; Tables S14 and S15). Seven of the proteins had a common overlap found with both controls and with that form the high confidence core of the Bre5-^C^Tb•Rps2-^N^Tb Split-TurboID experiment (Figure 6, proteins highlighted in green; Table S15). These seven proteins are the deubiquitinase Ubp3, the ubiquitin-associated factors Def1 and Lsm12 [17,41], Pbp1, the RNA helicase Ded1, Lsg1 (factor releasing *nonsense-mediated mRNA decay factor 3* from the 60S subunit, Nmd3 [42]), and the mRNA-binding protein Sro9. These results on the one hand confirm *hr40S* localization of proteins identified with the Asc1- and Rps-BioID experiments, and on the other hand verify transitional *hr40S* localization of the nucleus-active RNA polymerase II degradation factor Def1 and of the Pbp1-Pbp4-interacting protein Lsm12 [16,43].

## 3. Discussion

**Figure 7:**
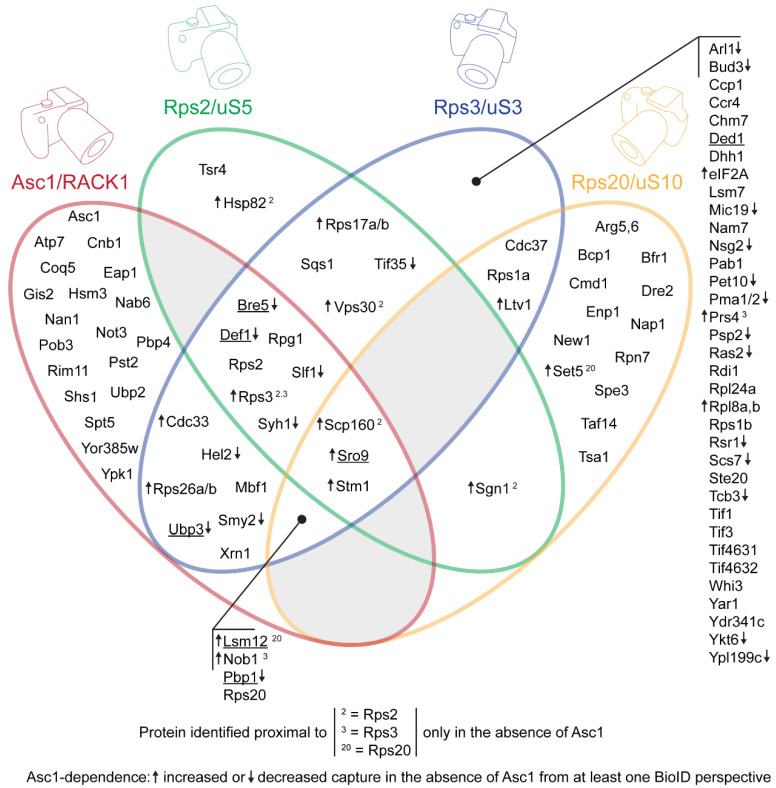
Four adjacent and overlapping perspectives at the *hr40S*: Comparison of microenvironments captured with Asc1-, Rps2-, Rps3-, and Rps20-BioID. The identified proteins co-localizing with the four different RPs are depicted within a Venn diagram. Proteins that were additionally identified in the common microenvironment of Bre5 and Rps2 with the Bre5-^C^Tb•Rps2-^N^Tb Split-TurboID are underlined.

Proximity of proteins not only occurs through direct physical binding, but also through transient common localization at or near larger structural components or machineries. Accurately localized bait-biotin ligases within the *hr40S* microenvironment covalently label proximal proteins in cell culture that are captured and identified afterwards from detergent-solubilized cell extracts. By taking different labeling positions within the *hr40S*, we have taken four pictures from this specific microenvironment from slightly shifted perspectives, namely from a biotin ligase fused to Asc1/RACK1, Rps2/uS5, Rps3/uS3, or Rps20/uS10 (Figure 7). Three non-ribosomal proteins appeared as common proximal factors of all four bait proteins, namely the mRNA-binding proteins Scp160 and Sro9, and the ribosome clamping factor Stm1. Common to these proteins is that they provide for mRNA tunnel occupation of the ribosomal 40S subunit, either with mRNA (through Scp160 and Sro9) or on its own (Stm1). The largest intersection set between two perspectives derived from Asc1-BirA* and Rps3-BirA*. This set contains 14 additional non-ribosomal proteins. Among them are the mRNA cap-binding protein Cdc33 and the a-subunit of eIF3 Rpg1, which reflects an mRNA 5′UTR-orientation of both Asc1 and the C-terminus of Rps3 at the *hr40S*. Slf1 is a paralog of the afore mentioned mRNA-binding protein Sro9. Ubiquitination, ubiquitin binding, and deubiquitination processes within that microenvironment are indicated by the proximity of the E3 RING ubiquitin ligase Hel2, the CUE domain- and poly(Q)-containing protein Def1, and the deubiquitination complex Bre5-Ubp3, respectively. The GYF-domain containing paralogs Smy2 and Syh1, and the mRNA 5′-3′ exonuclease Xrn1 hint to a broader translation-associated context of mRNA surveillance within that area. Mbf1, in concert with Asc1 and Rps3, has been described to prevent translational frameshifting at inhibitory CGA-CGA codon repeats [14]. The known interacting proteins Pbp1 and Lsm12, together with Pbp4 and Dhh1, are related to stress granules [44].

We also observed changes at the *hr40S* caused by the absence of Asc1, e.g., Hel2 and Bre5-Ubp3 were captured less (Figure 7). There are two possibilities for Asc1-dependent changes in protein captures with BioID: (i) Changes in the abundance of proteins at the *hr40S* or (ii) altered accessibility of proteins for biotinylation. The latter might be the case for Rps3 itself and for Rps26a/b in the Rps3-BioID experiment. The lysine residues within the C-terminal arm of Rps3 become increasingly accessible in the absence of Asc1 (Figure 1A), an observation that we had already made from the Rps2 perspective [18]. Shielding through Asc1 might also explain why Vps30, a subunit of the PI3K complexes I and II, was captured from the Rps2-BirA* perspective only in the absence of Asc1 [18], whereas it was captured independently of Asc1 from the Rps3-BirA* perspective.

The large number of proteins at the *hr40S* represents a cross-section of various microenvironments of context-specific ribosomes. Contexts can be specified by the presence of non-ribosomal proteins that co-localize at the *hr40S* only upon certain circumstances, e.g., early during ribosome biogenesis or later upon ribosome collision as a consequence of ribosome stalling. A first pioneering Split-TurboID experiment with Bre5 and the constitutive *hr40S* protein Rps2 provides a promising basis for studying distinct ribosomal microenvironments. In the following sections, we discuss the identified proteins within their, thus far, known contexts with regard to ribosome homeostasis and Asc1-dependence.

### 3.1. Regulation of eIF2α Phosphorylation by Gcn2 Might Require an Asc1-Dependent Positioning of Hel2 at hr40S

Asc1/RACK1 has an essential role in the recognition of translational stalling of ribosomes, e.g., on aberrant mRNAs during translation [45,46,47,48]. To prevent the synthesis of aberrant and toxic polypeptides and to take care of stuck polysomes, stalled ribosomes are recognized by the ribosome-associated protein quality control pathway (RQC). This pathway subjects the nascent polypeptide chain to proteasomal degradation [45]. Furthermore, ribosomal subunits need to be released and the mRNA degraded by endo- and exonucleolytic activities [49]. Ribosome stalling can induce collisions with trailing ribosomes and the formation of disomes [50,51]. The interface of such disomes is mainly formed by two 40S subunits and causes direct contact between the Asc1 molecules from the leading and the colliding ribosomes [50]. RPs within the concerned area are marked through ubiquitination. The E3-ubiquitin RING ligase Hel2 modifies Rps3/uS3 and Rps20/uS10 in *S. cerevisiae*, and its mammalian homolog ZNF598 ubiquitinates eS10 and uS10 [48,51,52,53,54]. Ubiquitination of RPs is followed by ribosomal subunit separation, the degradation of the associated nascent polypeptide chain and the aberrant mRNA, and by a recycling of ribosome subunits [50,51,54]. In mammalian cells, deubiquitination of RPs by the conserved Bre5-Ubp3 homologous deubiquitination complex G3BP1-family-USP10 is an important late step during 40S subunit recycling to prevent its lysosomal degradation [55]. In yeast, Bre5-Ubp3 also antagonizes the Hel2-mediated ubiquitination of Rps3 [30]. Our recent Asc1-BioID study revealed proximity of Asc1 to Hel2 and Bre5-Ubp3 [3]. With our new Rps3-BioID results, we confirmed an Asc1-dependent localization of Bre5-Ubp3 at the *hr40S*, which we had already observed earlier by studying the Rps2 perspective (Figure 7, [18]). The Rps3-BioID data further imply displacement of Hel2 in the absence of Asc1. An impaired ribosome localization of Hel2 has previously been reported for Asc1-deficient cells with polysome gradient experiments [56]. Thus, our findings underscore the importance of Asc1 for balancing ubiquitination and deubiquitination at the *hr40S*.

Recent studies have shown that ribosome collisions not only trigger translational quality control pathways, but also lead to broader cellular stress responses when they become more abundant and persistent [57,58,59]. The action of Hel2 during RQC prevents the activation of the integrated stress response (ISR) under low stress conditions when ribosome collisions are rare [59]. Under stress conditions and upon increased frequency of collisions, however, the eIF2α kinase Gcn2 is activated especially by ribosomes with an empty tRNA acceptor site [59]. eIF2α is a subunit of the heterotrimeric eIF2 complex. Phosphorylation of eIF2α by Gcn2 at S51 prevents formation of the ternary complex eIF2·GTP·Met-tRNA_i_, which is required for initiation of translation, and thus attenuates global translation [60,61]. Deletion of *HEL2* increases cellular eIF2α phosphorylation [59]. Previously, we observed elevated levels of eIF2α phosphorylation for an ∆*asc1* strain independent of applied stress conditions [9]. The impaired *hr40S* localization of Hel2 in Asc1-deficient cells and thus impairment of RQC might abrogate Hel2’s inhibitory effect on premature activation of Gcn2 under non-stress conditions leading to increased levels of phosphorylated eIF2α.

Similar to eIF2 the initiation factor eIF2A promotes Met-tRNA_i_ binding to the 40S subunit, yet, it is not required for global translation initiation and appears to be rather involved in specific initiation events, such as re-initiation, internal initiation, or initiation at non-AUG codons [62]. Here, we observed increased capture of eIF2A from the Rps3 microenvironment in the absence of Asc1. The observed increase in the phosphorylation of eIF2α in ∆*asc1* cells and the correlated activated ISR might cause the need for an alternative way to initiate translation that might be mediated by eIF2A.

### 3.2. The GYF Domain Proteins Smy2/Syh1, the Putative Endonuclease Ypl199c, and the 5′-3′ Exonuclease Xrn1 Localize to the hr40S

In this study, we also observed displacement of Smy2 at the *hr40S* in the absence of Asc1. Moreover, the capture of the Smy2 paralog Syh1 was Asc1-dependent in previous BioID experiments with Rps2-BirA* (Figure 7, [18]). Smy2 and Syh1 are potential homologs of mammalian GIGYF2, an inhibitor of translation [63,64]. Recent studies revealed that re-initiation of translation during RQC is prevented through the action of GIGYF2 and mRNA cap-binding protein 4EHP [65]. Their recruitment to faulty mRNAs seems to be mediated by different factors, including the Hel2 homolog ZNF598 [65] and the Mbf1 homolog EDF1 [15]. 4EHP is an eIF4E1 ortholog and inhibits initiation of translation through competition with eIF4E for cap-binding [66,67]. *S. cerevisiae* contains no obvious 4EHP homolog, and Smy2 and Syh1 seem to mediate mRNA decay rather than repression of translation during RQC [65]. However, Smy2 binds the eIF4E-binding protein Eap1, a translational repressor that competes with eIF4G for binding to eIF4E [68]. Smy2 and Eap1 together with Scp160 and Asc1 form the SESA network to regulate translation of *POM34* mRNA that encodes an integral component of the nuclear pore complex [68].

Here, for the first time, the yeast endonuclease Ypl199c was found proximal to Rps3. Ypl199c and Cue2 are two yeast endonucleases with homology to the *C. elegans* protein NONU-1 and have recently been described to have an evolutionarily conserved function in mRNA cleavage during nonstop and no-go mRNA decay [29,69]. In the absence of Asc1, Ypl199c enriched less from the Rps3-microenvironment. This might explain the previously reported importance of Asc1 for endonucleolytic cleavage of mRNA during nonstop decay [70]. Endonucleolytically cleaved mRNA is further degraded by the 5′-3′ exonuclease Xrn1 and the exosome [71]. Xrn1 localizes close to Asc1 [3,72] and was also captured here within the proximity of Rps3.

### 3.3. The hr40S Might Be Directly Linked to MAP Kinase Signaling through the MAP4K Ste20

We identified the MAP4K Ste20 as being proximal to Rps3 and thus, most likely, to translating ribosomes. In metazoa, an important sensor for colliding ribosomes is the MAP3K ZAKα [57,58]. The MAP3K associates with translating ribosomes and auto-phosphorylates itself when stable collisions become increasingly abundant and the quality control pathway gets overburdened [58]. Depending on the degree of ribosome collisions, ZAKα is required for the activation of GCN2 and stress-activated kinases p38 and JNK to reduce translation initiation and to induce apoptosis, respectively [58]. Similar to ZAKα, Ste20 might communicate the translation status to stress-activated kinases. Co-localization of Asc1 and Ste20 has been described earlier [6], and deletion of the *ASC1* gene causes increased phosphorylation of Ste20 at three different sites [73]. Phosphorylation of the downstream targets of Ste20-comprising MAPK cascades, the MAPKs Kss1 and the p38 homolog Hog1, increases and decreases upon *ASC1* deletion, respectively [6,73].

Overall, the *hr40S* seems to be sensitive to and dependent on the activity status of (poly)ribosomes and/or the context of their stalling, and thus might directly contribute to the adequate progressive, preservative or degradative processes. This encompasses cellular signaling by (de)ubiquitination and kinase-mediated protein phosphorylation, together regulating protein processing, structure remodeling, and protein trafficking. With that, ribosomes coupled to any specific cellular process might stall context-specifically (e.g., upon substrate depletion, stress, faulty mRNA) and simultaneously trigger the release of ribosome-bound factors destined for specific cellular target sites, such as mRNA binding proteins and Def1 for the nucleus. *hr40S* residence of Def1 was corroborated by the Bre5-^C^Tb•Rps2-^N^Tb Split-TurboID experiment, indicating the possibility that ribosome stalling or collision might trigger its ribosomal release and consecutive nuclear transfer. Indeed, it has been shown previously that ubiquitin-dependent chopping of a glutamine-rich C-terminal moiety of Def1 is essential for its nuclear accumulation [74]. Ongoing studies will address the hypothesis that signal and origin of the nuclear transfer of Def1 emerge from the *hr40S*, possibly coordinated by the scaffold protein Asc1.

## 4. Materials and Methods

### 4.1. Plasmid Construction

Plasmids used in this study are listed in Table 1. All new plasmids in this study were generated using the GeneArt™ Seamless Cloning and Assembly Kit (#10378809, Fisher Scientific GmbH, Schwerte, Germany), except for pME5058, which was generated with the In-Fusion HD Cloning Kit (#639650, Clontech Laboratories, Inc., Mountain View, CA, USA). To obtain plasmids pME4800 and pME5059-5061, the plasmid pME4478 was used as a template to generate the linearized vector backbone by PCR, and inserts were amplified from genomic *S. cerevisiae* DNA (RH2817), introducing the linker sequence and 15 bp overhangs complementary to the ends of the vector backbone. For the construction of pME5058, the insert contained 20 bp overhangs. Plasmids pME4984 (*^N^Tb-myc*) and pME4985 (*^C^Tb-myc*) are based on plasmids pME2785 and pME2787. The *^N^Tb* insert was amplified from pME4480 and the *^C^Tb* insert from a codon optimized template (provided by Hans Dieter Schmitt) [34,75]. For the *^N^Tb* insert, a codon exchange was introduced via the PCR primer to obtain the amino acid exchange Q65P. In the next step, pME4984 (*^N^Tb-myc*) and pME4985 (*^C^Tb-myc*) were used to amplify plasmid backbones for the construction of plasmids pME4986-4989 and pME5312. Inserts were amplified from genomic DNA and linker sequences were introduced via the oligonucleotides used for PCR. For plasmids pME4986 and pME4987, the *MET25* promoter was replaced by the native *ASC1* promoter [76]. Proteins were fused via four glycine-serine-serine (GSS) repeats as a linker to the BirA* protein, ^N^Tb, or ^C^Tb. The Rps3 linker contained two additional GSS repeats. Plasmid pME5307 was based on plasmid pFA6a-*VN*-*TRP1* [77]. The coding sequence for the N-terminal fragment of Venus (VN) was replaced by the *^C^Tb-myc* sequence and a linker sequence coding for the amino acid sequence GRRIPGLGSAGSAAGSGE was introduced.

### 4.2. Yeast Strains and Growth Conditions

Yeast strains used in this study are of the genetic ∑1278b background and listed in Table 2. Yeasts cells were cultivated in liquid yeast nitrogen base medium (YNB; 0.15% YNB, 0.5% ammonium sulfate, 2% glucose). L-arginine (20 mg/L), L-lysine HCl (30 mg/L), L-tryptophan (20 mg/L), and L-proline (200 mg/L) were added as required. To obtain strain RH3902 for the expression of Bre5-^C^Tb-myc, the transformation cassette was amplified from plasmid pME5307. Primers used for PCR contained overhangs identical to the target site for homologous recombination. The transformation cassette was inserted into the genome of strain RH2817.

### 4.3. Western Blot Analysis

Cell lysis was performed as described in Schmitt and Valerius (2019) [18] according to Kushnirov (2000) [79]. For each sample, an equal number of yeast cells (calculated according to the OD_600_ of the culture, e.g., 6.25 mL of culture with OD_600_ of 0.8) was harvested by centrifugation. Pelleted cells were washed with water and incubated in 0.1 M NaOH for 5 min at room temperature. NaOH was removed and yeast cells were resuspended in 100 µL of 1:4 diluted SDS-PAGE loading buffer (0.25 M Tris pH 6.8, 30% glycerol, 15% β-mercaptoethanol, 7% SDS, 0.3% bromphenol blue). Samples were incubated at 95 °C for 3 min and centrifuged at 13,000 rpm for 5 min. Proteins of 6 µL of the supernatant were separated by SDS-PAGE, blotted onto a nitrocellulose membrane and subsequently stained with Ponceau S (0.2% Ponceau S, 3% trichloroacetic acid). After blocking of the membranes with 5% milk powder in tris buffered saline (TBS; 20 mM Tris, 150 mM NaCl), the membranes were incubated with a BirA antibody (#orb230654, biorbyt), c-myc antibody (#9E10, Santa Cruz SC40), or Rps3 antibody (provided by Heike Krebber, [3]), followed by incubation with the peroxidase-coupled goat anti-rabbit antibody. For the detection of biotinylated proteins, membranes were blocked overnight with 1% BSA in phosphate buffered saline (PBS, 8 mM Na_2_HPO_4_, 2 mM NaH_2_PO_4_, 150 mM NaCl) and incubated with Pierce^TM^ High Sensitivity Streptavidin-HRP (#21130, Thermo Fisher Scientific, Waltham, Massachusetts, USA) diluted 1:2000 in the blocking buffer with 0.1% Tween 20) for 1 h at room temperature. The FUSION-SL-4 (Peqlab Biotechnology GmbH, Erlangen, Germany) was used for the detection of chemiluminescent signals.

### 4.4. Proximity Labeling MS Experiments

Yeast cells were grown in 200 mL (Rps3-BioID experiment) or 100 mL (Rps20-BioID and Split-TurboID experiment) YNB medium containing 10 µM D-biotin to mid-log phase. For the Split-TurboID experiment, biotin was added when the cells reached an OD_600_ of 0.25 and cells were harvested after 3 h incubation. The media additionally contained differentially labeled lysine and arginine for SILAC (see Figure 2A and Figure 5D for labeling of the strains). ^13^C_6_-L-arginine HCl, ^13^C_6_^15^N_4_-L-arginine HCl, 4,4,5,5-D_4_-L-lysine HCl, ^13^C_6_-L-lysine HCl were used for the labeling of the strains with *medium* and *heavy* amino acids. For the Split-TurboID experiment, L-proline was additionally added. After harvesting the cells, equal amounts of cells from each culture were mixed according to the OD_600_ of the main cultures. Cells were washed with washing buffer 1 (10 mM HEPES, pH 7.9, 10 mM KCl, 1.5 mM MgCl_2_) and lysed in 1.8 mL breaking buffer (wash buffer 1 with 1 cOmplete^TM^ EDTA-free protease inhibitor cocktail tablet (#5056489001, Merck) per 50 mL, 0.5 mM DTT and 0.5 mM PMSF) using glass beads, and SDS was added to a final *w/v* of 4%. For the enrichment of biotinylated proteins in the Rps3-BioID experiments, Strep-Tactin^®^ Sepharose^®^ gravity flow columns with 0.2 mL bed volume were used (#2-1202-550, IBA Lifesciences GmbH, Göttingen, Germany) as described in Schmitt and Valerius (2019) [18]. For the enrichment of biotinylated proteins in the Rps20-BioID and the Split-TurboID experiments, 200–300 µL Strep-Tactin^®^ Superflow resin (#2-1206-025, IBA Lifesciences GmbH) were used instead. Due to the smaller culture volume, cells were lysed in only 0.9 mL breaking buffer. The Strep-Tactin^®^ beads were washed twice with 750 µL washing buffer 2 (0.1 M Tris-Cl, 150 mM NaCl, 1 mM EDTA, #2-1003-100, IBA Lifesciences GmbH) for equilibration. The cell lysate was added to the equilibrated beads and incubated for 30 min with gentle rotation. The samples were washed three times with 1 mL washing buffer 2 containing 0.4% *w*/*v* SDS. Biotinylated proteins were eluted through incubation of the beads with 150 µL BXT buffer (0.1 M Tris-Cl, 150 mM NaCl, 1 mM EDTA, 50 mM biotin #2-1042-025, IBA Lifesciences GmbH) for 10 min. The elution step was repeated once, and proteins were isolated from the eluate by chloroform-methanol extraction [80]. Protein pellets were reconstituted in 20 µL 8 M urea/2 M thiourea and subjected to SDS-PAGE followed by incubation in fixing solution (40% ethanol, 10% acetic acid) for at least 1 h. As input control samples, 50 µg of proteins from the whole cell lysate were separated by SDS-PAGE. Complete gel lanes were divided in ten fractions/gel slices, the proteins digested within the gel with trypsin, and peptides were extracted [81]. To evaluate the labeling efficiency for the Split-TurboID experiment, protein lysates were prepared as described in 4.3 using aliquots of the still separate cell cultures. A total of 6 µL of each cell lysate was used for SDS-PAGE. One gel slice was cut out from the gel lane and used for in-gel digest of proteins with trypsin. Peptides were purified using C18 (#2215, 3M) stop and go extraction (stage) tips [82,83]. Peptides were dried, dissolved in 20 µL sample buffer (2% acetonitrile, 0.1% formic acid), and analyzed through LC-MS using an UltiMate 3000 RSLCnano system coupled to either an Orbitrap Velos Pro hybrid ion trap-Orbitrap or a Q Exactive HF mass spectrometer (all Thermo Fisher Scientific). For the first replicate of the Rps20-BioID, the samples from the eluate fractions were measured twice. SILAC ratios for identified proteins were later on averaged for these two technical replicates. LC-MS analysis was performed as described before [18]. LC-MS method programming and data acquisition were performed with the software XCalibur 2.2 (Thermo Fisher Scientific). MS/MS2 data were searched against an *S. cerevisiae*-specific protein database (UniProt Proteome ID UP000002311) additionally containing the amino acid sequence of *Escherichia coli* BirA (UniProtKB: P06709) or of ^N^Tb and ^C^Tb [34] using the software MaxQuant 1.6.12.0 [22]. With respect to the SILAC labeling of the yeast cultures, Arg6 and Lys4 alongside Arg10 and Lys8 were defined as the *medium* and *heavy* labels, respectively. SILAC ratios for BirA* were later not further considered due to the different molecular weight of the BirA* fusion proteins, and thus, their distribution in different fractions. Also, SILAC ratios for ^N^Tb and ^C^Tb were removed. Biotinylation of lysine was set as variable modification in addition to oxidation of methionine and N-terminal acetylation. Re-quantification was enabled and a minimum of one ratio count was required for protein quantification using unique and razor peptides. For all other parameters, the default settings were used. MaxQuant output data were analyzed using the Perseus software 1.6.0.7 [23].

### 4.5. Phenotypic Growth Test

Yeast cells were cultivated to mid-log phase in YNB medium and diluted to an OD_600_ of 0.1. Three tenfold dilutions were prepared, and 20 µL of each dilution were spotted on the agar plates containing either 10 µM biotin or 0.05 µg/mL cycloheximide. A plate without additional supplements was used for the growth control. Plates were incubated at 30 °C for 3 to 4 days.

## Figures and Tables

**Table 1:** Plasmids used in this study.

Plasmid Name	Description	Reference
pME2785	*MET25Prom*, *CYC1Term*, *TRP1*, 2 μm	[78]
pME2787	*MET25Prom*, *CYC1Term*, *URA3*, 2 μm	[78]
pME4480	*MET25Prom*, *CYC1Term*, *URA3*, 2 μm, birA*	[3]
pME4478	*MET25Prom*, *CYC1Term*, *URA3*, 2 μm, ASC1-birA*	[3]
pME4800	*MET25Prom*, *CYC1Term*, *URA3*, 2 μm, RPS20-birA*	This study
pME5058	*MET25Prom*, *CYC1Term*, *URA3*, 2 μm, RPS3-birA*	This study
pME5059	*MET25Prom*, *CYC1Term*, *URA3*, 2 μm, FBA1-birA*	This study
pME5060	*MET25Prom*, *CYC1Term*, *URA3*, 2 μm, RPL5-birA*	This study
pME5061	*MET25Prom*, *CYC1Term*, *URA3*, 2 μm, RPL25-birA*	This study
pME4984	*MET25Prom, CYC1Term*, *URA3*, 2 μm, *^N^Tb-myc*	This study
pME4985	*MET25Prom, CYC1Term*, *TRP1*, 2 μm, *^C^Tb-myc*	This study
pME4986	*natProm^ASC1^, CYC1Term*, *URA3*, 2 μm, *ASC1-^N^Tb-myc*	This study
pME4987	*natProm^ASC1^, CYC1Term*, *TRP1*, 2 μm, *ASC1-^C^Tb-myc*	This study
pME4988	*MET25Prom, CYC1Term*, *URA3*, 2 μm, *RPS2-^N^Tb-myc*	This study
pME4989	*MET25Prom, CYC1Term*, *TRP1*, 2 μm, *RPS2-^C^Tb-myc*	This study
pFA6a-*VN-TRP1*	pFA6a, *VN*, *TRP1*, *ADH1Term*	[77]
pME5307	pFA6a, *^C^Tb-myc*, *TRP1*, *ADH1Term*	This study
pME5312	*MET25Prom, CYC1Term*, *URA3*, 2 μm, *RPL5-^N^Tb-myc*	This study

**Table 2:** *S. cerevisiae* strains used in this study.

Strain Name	Description	Reference
RH2817	*MATα*, *ura3-52*, *trp1::hisG*	[9]
RH3510	*MATα*, *ura3-52*, *trp1::hisG*, *asc1-loxP SNR24*	[8]
RH3493	*MAT*α, *ura3-52, trp1::hisG,* Δ*arg4::loxP,* Δ*lys1::loxP*	[73]
RH3520	*MATα*, *ura3-52*, *trp1::hisG*, *asc1-loxP SNR24*, ∆*arg4::loxP*, ∆*lys1::loxP*	[73]
RH3789	*MATα*, *ura3-52*, *trp1::hisG,* Δ*bre5::kanMX4*	[18]
RH3902	*MATα*, *ura3-52*, *trp1::hisG, BRE5-^C^Tb-myc::TRP1*	This study

## Data Availability

The mass spectrometry proteomics data have been deposited to the ProteomeXchange Consortium via the PRIDE partner repository with the dataset identifiers PXD027267 and PXD028879 [84,85].

## Supplementary Materials

The following are available online at https://www.mdpi.com/article/10.3390/ijms222111653/s1.
