# Supplementary material for: A Multi-Perspective Proximity View on the Dynamic Head Region of the Ribosomal 40S Subunit
Source: Int J Mol Sci. 2021 Oct 28;22(21):11653. doi: 10.3390/ijms222111653 (PMC8583833; doi:10.3390/ijms222111653)
Supplement: Supplementary file 1 [file ijms-22-11653-s001.zip › ijms-1359139-supplementary/Table S1_S10_S11_S12.pdf]

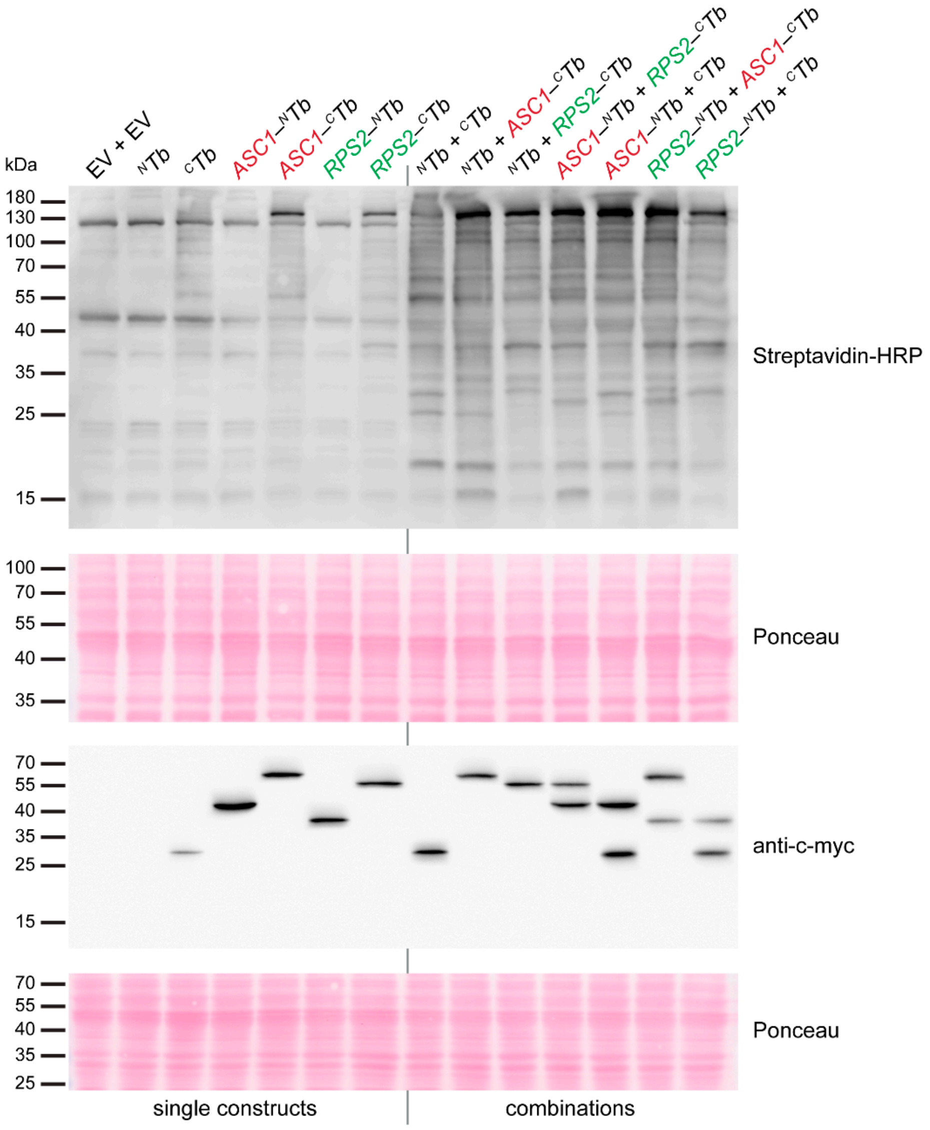

Figure S1. Expression and activity of Split-TurboID constructs. Free ^{N}Tb and ^{C}Tb and their fusion proteins with Asc1 and Rps2 were expressed individually and in different combinations (labeled 'single constructs' and 'combinations' at the bottom). The yeast cells were incubated for 1 h with biotin. A Western blot with cell lysates using a c-myc-specific antibody is shown. Due to its small size, ^{N}Tb (10 kDa) was not detected. Streptavidin-HRP was used to detect biotinylated proteins. ^{N}Tb expression is confirmed through its complementing contribution to biotinylation activity of ^{C}Tb constructs. Ponceau staining of proteins on the membranes served as a loading control.

Table S1: Perseus analysis workflow of the MaxQuant database search result for Rps3-BioID LC-MS data.

For the Rps2-BioID experiment the following analysis workflow was performed accordingly. The protein SILAC ratios from two LC-MS measurements of technical replicates of the first Rps2-BioID enrichment fractions were averaged in Perseus.

No.	Command	Description
1	Generic matrix upload	proteinGroups.txt, select normalized ratios etc.
2.1	Filter rows based on categorical column	Remove rows with "+" in reverse column
2.2		Remove rows with "+" in potential contaminant column
2.3		Remove rows with "+" in only identified by site column
3	Transform	Inverse ratios M/L (1/x), ratios are reported as follows:

		*ASC1 RPS3-birA**/ *ASC1 RPL5-birA** (L/M)<br>*asc1*$^-$ *RPS3-birA**/ *ASC1 RPS3-birA** (H/L)<br>*asc1*$^-$ *RPS3-birA**/*ASC1 RPL5-birA** (H/M)
4	Transform	$\log_2(x)$
5	Categorical annotation rows	Group all nine enrichment ratios → “enrichment”
**Proteins with 9 valid enrichment ratios**		
6	Filter rows based on valid values	9 values in grouping “enrichment” (see step 5) valid, reduce matrix
7	Replace missing values by constant	Replace missing values in proteome (P) samples with value 0
8	Combine main columns	enrichment (E) ratios minus proteome ratios (P)<br>→ proteome corrected ratios E-P
**Filter for proteins enriched from *ASC1 RPS3-birA****		
9	One-sample tests	Select ratios E-P *ASC1 RPS3-birA**/ *ASC1 RPL5-birA** (L/M)<br>threshold p-value = 0.05
10	Filter rows based on numerical/ main column	Filter t-test difference ≥ 0.585<br>Add categorical column (keep or discard)
11	Combine categorical columns	Combine t-test significant and filter
12	Filter rows based on categorical column	Keep rows with “+_Keep” in combined categorical column
13	Replace imputed values by NaN	Imputed values (see step 7) are replaced by NaN
**Filter for proteins differentially enriched from *asc1*$^-$ *RPS3-birA** and *ASC1 RPS3-birA****<br>**Proceed from step 8 with the following steps (14-20)**		
14	One-sample tests	Select ratios E-P *asc1*$^-$ *RPS3-birA**/ *ASC1 RPS3-birA** (H/L)<br>threshold p-value = 0.05
15	Filter rows based on numerical/ main column	Filter 1: t-test difference ≥ 0.585 or ≤ -0.585<br>Add categorical column (keep or discard)
16	Categorical annotation rows	Grouping *E1 and E2* with two groups:<br>E1: E-P *ASC1 RPS3-birA**/ *ASC1 RPL5-birA** (L/M)<br>E2: E-P *asc1*$^-$ *RPS3-birA**/ *ASC1 RPL5-birA** (H/M)
17	Filter rows based on valid values	3 valid values in grouping *E1 and E2*: greater than or equal to 0.585<br>Add categorical column → filter 2
18.1<br>18.2	Combine categorical columns	Combine *t-test significant* and *filter 1*<br>Combine *t-test significant_filter 1* and *filter 2*
19	Filter rows based on categorical column	Filter rows based on combined columns<br>*significant_filter 1_filter 2*<br>Keep rows matching “+_keep_keep”
20	Replace imputed values by NaN	Imputed values (see step 7) are replaced by NaN

Table S1. Continued

**Proteins with only 6 valid enrichment ratios - Proceed from step 5 with the following steps**		
21	Filter rows based on valid values	6 values in grouping “enrichment” (see step 5) valid, reduce matrix
22	Filter rows based on valid values	9 values in grouping “enrichment” (see step 5) valid, Add categorical row
23	Filter rows based on categorical column	Remove rows with “keep” (see step 22)
24	Categorical annotation rows	Group all replicates

25	Average groups	Average groups (see step 24), calculate mean, keep original data
26	Change column type	Change numerical columns with mean values into main columns
27	Replace missing values by constant	Replace missing values by 0 for averaged proteome ratios
28	Combine main columns	Averaged enrichment ratios minus respective proteome ratios → Averaged E-P
**Filter for proteins enriched from *ASC1 RPS3-birA****		
29	Filter rows based on numerical/ main column	Filter proteome-corrected ratios *ASC1 RPS3-birA*/ASC1 RPL5-birA** (see step 28) for 1 valid value ≥ 0.585
30	Categorical annotation rows	Group enrichment ratios *ASC1 RPS3-birA*/ASC1 RPL5-birA**
31	Filter rows based on valid values	2 values in group *ASC1 RPS3-birA*/ASC1 RPL5-birA** ≥ 0.585
32	Replace imputed values by NaN	Imputed values (see step 7) are replaced by NaN
33	Filter rows based on numerical/ main column	Filter for proteins with in total ≥ 5 M/L ratios counts in enrichment samples
**Filter for proteins differentially enriched from *asc1*$^{-}$ *RPS3-birA** and *ASC1 RPS3-birA**<br>Proceed from step 28 with the following steps (34-41)**		
34	Filter rows based on numerical/ main column	Filter proteome-corrected ratios *asc1*$^{-}$ *RPS3-birA*/ASC1 RPS3-birA** (see step 28) for 1 valid values ≥ 0.585 or ≤ -0.585
35	Filter rows based on numerical/ main column	Filter proteome-corrected ratios *ASC1 RPS3-birA*/ASC1 RPL5-birA** and *asc1*$^{-}$ *RPS3-birA*/ASC1 RPL5-birA** (see step 28) for at least 1 valid value ≥ 0.585
36	Categorical annotation rows	Group enrichment ratios *asc1*$^{-}$ *RPS3-birA*/ASC1 RPS3-birA**
37	Filter rows based on valid values	2 values in group *asc1*$^{-}$ *RPS3-birA*/ASC1 RPS3-birA**<br>≥ 0.585<br>Add categorical column (keep or discard)
38	Filter rows based on valid values	2 values in group *asc1*$^{-}$ *RPS3-birA*/ASC1 RPS3-birA**<br>≤ -0.585<br>Add categorical column (keep or discard)
39	Combine categorical columns	Combine categorical columns generated through step 37 and 38
40	Filter rows based on categorical column	Keep rows with combined filter “Keep_Discard” or “Discard_Keep”
41	Filter rows based on numerical/ main column	Filter for proteins with in total ≥ 5 H/L ratio counts in enrichment samples

Table S10. Perseus workflow for the evaluation of the labeling efficiency.

No.	Command	Description
1	Generic matrix upload	msms.txt, use default selection and "missed cleavage sites"
2	Filter rows based on categorical column	Remove rows with "+" in reverse column
3	Filter rows based on text column	Filter for "CON_" in column "Proteins", remove matching rows, reduce matrix
4	Filter rows based on numerical column	Number of missed cleavage sites = 0
5	Filter rows based on categorical column	Filter for raw file number
6	Filter rows based on categorical column	Filter for unlabeled peptides for "light" samples and filter for Lys4/Arg6 and Lys8/Arg10 labeled peptides, respectively, for the "medium" and "heavy" samples

Table S11. 2nSILAC labeling efficiency for the Bre5-^{C}Tb•Rps2-^{N}Tb Split-Tb experiment.
Only peptides (MSMS counts) without missed cleavage sites were considered (see Table S2). The total number of MSMS counts for each sample (replicates 1-3 = Rep. 1-3) is listed. For the "light" samples the number of MSMS counts for peptides containing no SILAC label is given as "correctly labeled", for the "medium" and "heavy" samples the MSMS counts for the respectively labeled peptides is provided. The percentage of correctly labeled peptides is given as the labeling efficiency.

	Light *BRE5-^{C}Tb + RPS2-^{N}Tb*			Medium *BRE5-^{C}Tb* + EV			Heavy *BRE5-^{C}Tb + RPL5-^{N}Tb*		
	Rep. 1	Rep. 2	Rep. 3	Rep. 1	Rep. 2	Rep. 3	Rep. 1	Rep. 2	Rep. 3
Total number MSMS counts	5377	5513	7672	4688	7181	8050	5576	5947	5682
Number MSMS counts for correctly labeled peptides	5375	5510	7662	4639	7079	7946	5520	5856	5608
Labeling efficiency [%]	99.96	99.95	99.87	98.95	98.58	98.71	99.00	98.47	98.70

Table S12. Perseus analysis workflow of the MaxQuant database search result for the Bre5-Rps2-Split-Tb LC-MS data.

No.	Command	Description
1	Generic matrix upload	proteinGroups.txt, select normalized ratios etc.
2.1	Filter rows based on categorical column	Remove rows with “+” in reverse column
2.2		Remove rows with “+” in potential contaminant column
2.3		Remove rows with “+” in only identified by site column
3	Transform	Inverse ratios M/L and H/L (1/x), ratios are reported as follows:<br>*BRE5-^{C}Tb•RPS2-^{N}Tb / BRE5-^{C}Tb*•EV (L/M)<br>*BRE5-^{C}Tb•RPS2-^{N}Tb / BRE5-^{C}Tb•RPL5-^{N}Tb* (L/H)
4	Transform	$\log_2(x)$
5	Categorical annotation rows	Group all 6 enrichment ratios → “enrichment”
6	Filter rows based on valid values	6 values in grouping “enrichment” (see step 5) valid, reduce matrix
7	Replace missing values by constant	Replace missing values in proteome (P) samples with value 0
8	Combine main columns	enrichment (E) ratios minus proteome ratios (P)<br>→ proteome corrected ratios E-P
9	Filter rows based on numerical/ main column	Peptides E_01 > 1<br>Peptides E_02 > 1<br>Peptides E_03 > 1
**Filter for proteins differentially enriched from *BRE5-^{C}Tb•RPS2-^{N}Tb* microenvironment using the *BRE5-^{C}Tb*•EV control**		
10	One-sample tests	Select ratios E-P *BRE5-^{C}Tb•RPS2-^{N}Tb / BRE5-^{C}Tb*•EV (L/M)<br>threshold p-value = 0.05
11	Filter rows based on numerical/ main column	Filter t-test difference ≥ 0.585<br>Add categorical column (keep or discard)
12	Combine categorical columns	Combine t-test significant and filter
13	Filter rows based on categorical column	Keep rows with “+_Keep” in combined categorical column
14	Replace imputed values by NaN	Imputed values (see step 7) are replaced by NaN
**Filter for proteins differentially enriched from *BRE5-^{C}Tb•RPS2-^{N}Tb* microenvironment using the *BRE5-^{C}Tb•RPL5-^{N}Tb* control**<br>**Proceed from step 9 with the following steps (15-19)**		
15	One-sample tests	Select ratios E-P *BRE5-^{C}Tb•RPS2-^{N}Tb / BRE5-^{C}Tb•RPL5-^{N}Tb* (L/H)<br>threshold p-value = 0.05
16-19	see step 11-14	
